# Sensitivity and specificity of ultrasonography in diagnosing supraspinatus lesions: a prospective accuracy diagnostic study

**DOI:** 10.1590/1516-3180.2018.0069170418

**Published:** 2018-08-13

**Authors:** João Alberto Yazigi, Fábio Anauate Nicolao, Fabio Teruo Matsunaga, Nicola Archetti, Marcelo Hide Matsumoto, Marcel Jun Sugawara Tamaoki

**Affiliations:** I MD. Doctoral Student and Attending Physician in the Shoulder and Elbow Surgery Clinic, Department of Orthopedics and Traumatology, Escola Paulista de Medicina (EPM), Universidade Federal de São Paulo (Unifesp), São Paulo, Brazil.; II MD. Doctoral Student and Attending Physician in the Shoulder and Elbow Surgery Clinic, Department of Orthopedics and Traumatology, Escola Paulista de Medicina (EPM), Universidade Federal de São Paulo (Unifesp), São Paulo, Brazil.; III MD, PhD. Attending Physician in the Shoulder and Elbow Surgery Clinic, Department of Orthopedics and Traumatology, Escola Paulista de Medicina (EPM), Universidade Federal de São Paulo (Unifesp), São Paulo, Brazil.; IV MD, PhD. Affiliated Professor and Head of the Shoulder and Elbow Surgery Clinic, Department of Orthopedics and Traumatology, Escola Paulista de Medicina (EPM), Universidade Federal de São Paulo (Unifesp), São Paulo, Brazil.; V MD, PhD. Attending Physician in the Shoulder and Elbow Surgery Clinic, Department of Orthopedics and Traumatology, Escola Paulista de Medicina (EPM), Universidade Federal de São Paulo (Unifesp), São Paulo, Brazil.; VI MD, PhD. Adjunct Professor, Department of Orthopedics and Traumatology, Escola Paulista de Medicina (EPM), Universidade Federal de São Paulo (Unifesp), São Paulo, Brazil.

**Keywords:** Rotator cuff, Magnetic resonance imaging, Ultrasonography

## Abstract

**BACKGROUND:**

This study was designed to define the accuracy of shoulder ultrasonography for diagnosing supraspinatus tendon tears. This examination is routinely used by orthopedists and may do away with the need for other examinations for diagnosing these tendon injuries. The aim of this study was to evaluate the sensitivity and specificity of shoulder ultrasonography for diagnosing supraspinatus tendon injuries, using magnetic resonance imaging as the reference.

**DESIGN AND SETTING:**

Prospective accuracy study at a single center: the Shoulder and Elbow Surgery Clinic of the Department of Orthopedics and Traumatology.

**METHODS:**

Shoulder ultrasonography was performed on 80 patients of both genders, over 18 years of age, with complaints of shoulder pain and clinically suspected supraspinatus tendon lesions. Jobe’s test and a full can test were performed. In addition, they underwent magnetic resonance imaging in a 3.0-tesla machine, as the reference standard. The examinations were performed and interpreted by radiologists.

**RESULTS:**

Ultrasonography showed sensitivity of 36.3% and specificity of 91.7% for supraspinatus tears overall: sensitivity of 25.8% and specificity of 91.8% for partial tears and sensitivity of 46.2% and specificity of 100% for full-thickness tears. Ultrasonography showed high accuracy for diagnosing full-thickness tears: 91.3%. The p-values were 0.003 for tears overall, 0.031 for partial tears and < 0.001 for full-thickness tears.

**CONCLUSIONS:**

Ultrasonography showed low sensitivity for detecting supraspinatus tears, but high specificity for both partial and full-thickness tears.

## INTRODUCTION

Rotator cuff tears (RCTs) are the main cause of shoulder pain in adults and the supraspinatus tendon is the element most affected.^1^^,^^2^ Studies have shown that the prevalence of RCTs ranges from 5% to 40% and that it is directly related to increasing age.^3^

Clinical tests and imaging examinations are routinely performed to diagnose lesions of this tendon.^3^ In a study conducted to elucidate the prevalence of rotator cuff tears in the general population, using ultrasonography as the reference standard, Yamamoto et al. found that the prevalence of rotator cuff tears was 20.7%, through examining 683 patients (total of 1,366 shoulders). They showed that the frequency of rotator cuff tears increased with age and that these lesions were most common in elderly male patients.^4^

Ultrasonography of the shoulder is a diagnostic method used in clinical practice by orthopedists. It is a non-invasive method that is accessible for most patients (both in primary and in tertiary-level healthcare services). It has low cost and high acceptability and allows viewing of rotator cuff tendons.^5^ However, it is a diagnostic method with potential risks of pitfalls, depending on the examiner’s technique and experience.^6^

Several studies assessing the accuracy of ultrasonography have been published. However, the literature still presents inconsistencies and variability regarding the sensitivity and specificity of this test in making the diagnosis of rotator cuff lesions.^7^ It has been shown that the sensitivity and specificity of ultrasonography are very similar to those of magnetic resonance imaging in diagnosing supraspinatus lesions, but orthopedists have reported some discrepancies in clinical practice.^8^

In our orthopedic practice, it is common to find disagreements between magnetic resonance and ultrasonography in making the diagnosis of supraspinatus tendon tears. Thus, the objective of this study was to assess the accuracy of shoulder ultrasonography for diagnosing tears of the supraspinatus tendon, taking magnetic resonance imaging of the shoulder as a reference standard.

## METHODS

### Study design, setting and ethics

We conducted a prospective accuracy study that included adult patients followed up at a single center, the Shoulder and Elbow Surgery Clinic of the Department of Orthopedics and Traumatology, Federal University of São Paulo (Universidade Federal de São Paulo, UNIFESP), between November 2016 and May 2017. This project was approved by our institution’s Research Ethics Committee under the number 0557/2017. All patients included had previously agreed to participate and had signed a written consent form, after being informed about the prognosis for and potential complications of their condition and the objectives of the study. We used the STARD checklist to guide the information in this article.

### Participants

The inclusion criteria were that the patients could be of either gender, needed to be over 18 years of age and had presented complaints of shoulder pain for at least one month. The exclusion criteria were occurrences of loss of passive movement of the shoulder (severe osteoarthrosis or adhesive capsulitis); sensory and motor deficits of the affected limb; fractures, dislocations, neoplastic lesions or previous surgery on the affected limb; absence of the results from the imaging tests (ultrasonography and magnetic resonance imaging); and time between ultrasonography and magnetic resonance imaging of over three months.

We recruited patients based on their presentation of symptoms of shoulder pain. These patients came for medical appointments at the shoulder and elbow clinic between November 2016 and May 2017. At our institution, there is a state-level referral center for shoulder diseases, for patients with referrals from this hospital and from other regions of the city and state. The patients included in this study were evaluated clinically using special tests for supraspinatus lesions (Jobe’s test and the full can test), and after the ultrasound examination (index test) had been performed by a radiologist belonging to the hospital team, magnetic resonance imaging (reference standard) was requested.

### Ultrasound and magnetic resonance evaluation methods

A total of five radiologists participated in the ultrasound and magnetic resonance imaging (MRI) evaluations, as specified below.

Ultrasound examination (index test): The ultrasonographic evaluation on each patient was performed by one of two radiologists who were not musculoskeletal specialists. A single ultrasound examination was performed per patient. Imaging was produced in the coronal, axial and sagittal planes, using a 10-MHz linear transducer.

Magnetic resonance imaging (reference standard): another three radiologists reported on the MRI examinations, i.e. not the same ones who performed the ultrasound examinations. Each patient was positioned in horizontal dorsal decubitus, with slight elevation of the unaffected shoulder. The arm of the affected side was kept alongside the body in slight external rotation. The affected shoulder was positioned as close as possible to the center of the magnet. Three acquisition planes were imaged, using T2 weighting with saturation of the fat signal:

- Axial plane from the apex of the acromioclavicular joint to the lower recess of the glenohumeral joint;
- Coronal oblique plane parallel to the supraspinatus and covering the scapulohumeral joint;
- Oblique sagittal plane perpendicular to the supraspinatus, from the distal end of the tendon to the middle of the rotator cuff muscle belly.

Using T2 weighting, 16 to 20 slices were obtained from each acquisition plane, in which the thickness was less than or equal to 4 mm, with a gap of the order of 10%. Two T1-weighted planes were imaged without saturation of the fat signal, with thicknesses of 4 mm to 5 mm and centered on the rotator cuff muscles:

- Oblique coronal plane: 12 to 16 slices, parallel to the supraspinatus and covering the scapulohumeral joint;
- Sagittal plane: 12 to 16 slices, covering from the tuberosity to the medial third of the scapula.

### Examination review and variables

The lesions were characterized in terms of the presence of tendinopathy and partial or full-thickness tears of the supraspinatus tendon. The radiologists took the following to be indicative of tears: absence of tendon visualization on ultrasonography; and hypersignal on all T2-weighted slices of the MRI of the shoulder.

The ultrasonography and magnetic resonance imaging of the shoulders that were performed by the radiologist team (formed by five radiologists: two who performed ultrasonography and three who performed MRI) were assessed. These radiologists were experienced medical staff members who were accustomed to performing these examinations but were not specialists in musculoskeletal disorders. There was no communication between the two radiologists who performed US and the other three who performed MRI. The diagnoses were made at the radiologists’ discretion and no previous training was provided. Thus, they detected lesions in accordance with their own learning and experience. The time that elapsed between ultrasonography and magnetic resonance imaging was a maximum of three months, and the MRI was always performed on another day and in another place, after the ultrasonography.

The lesions of the supraspinatus tendon were classified as:

1. Tendinopathy;
2. Presence of tears:
  1. A. partial tear; (alpha-upper)
  2. B. full-thickness tear.

### Statistical analysis

After collecting the data, we drew up double-entry tables of the ultrasound results as a function of the magnetic resonance results and then we calculated the sensitivity, specificity, positive and negative predictive values, positive and negative likelihood ratios and accuracy. The significance was assessed at the 5% level and the chi-square test was used to find the significance of the study parameters on a categorical scale between two or more groups.

The sample size was calculated as follows:

It was firstly estimated that the target population would be 96 patients over the study period, based on previous numbers of monthly visits to our institution’s outpatient clinic, which formed the source for our subjects. We assumed a sampling error of approximately 5% and took a 95% confidence interval. We then used the formula contained in the following website to calculate the sample size: https://pt.surveymonkey.com/mp/sample-size-calculator. In this manner, we found that the total sample size would need to be 77 patients. Based on the central limit theorem and the laws of large numbers, this sample size was sufficient to ensure that the statistical analyses would be reliable.

## RESULTS

Eighty-five patients were attended at the Shoulder and Elbow Surgery Sector between November 2016 and May 2017. Five were excluded because their examination results were not available (ultrasonography or magnetic resonance imaging). There were no adverse events from performing the index tests or the reference standard. The ultrasonography and magnetic resonance imaging were performed with a maximum of three months between them. The patients who were excluded were referred to the shoulder and elbow clinic for clinical reassessment. Out of the 80 patients assessed, 51 (63.75%) were male and 29 (36.25%) female. The male-to-female ratio was 1.75:1. The ages of the participants ranged from 19 to 72 and the mean age was 48.9 ± 2.5 years. The mean length of time with shoulder pain symptoms was 33.9 ± 11.4 months. There were six patients (7.5%) with a history of smoking. Nine (11.25%) of the patients had suffered trauma. The right side was affected in 47 of the participants (58.75%) and the left side in 33 (41.25%); the dominant side was affected in 53 (66.3%) of them (Table 1**)**.

**Table 1:** Study patients’ characteristics

Gender	Male	n = 51 (63.75%)
	Female	n = 29 (36.25%)
Age	Average age	48.9 ± 2.5 years
Duration of shoulder pain	Average	33.9 ± 11.4 months
Side	Right	n = 47 (58.75%)
	Left	n = 33 (41.25%)
Dominance	Dominant side	n = 53 (66.3%)
	Non-dominant side	n = 27 (33.8%)
Supraspinatus lesions as detected using magnetic resonance imaging	No injuries	n = 6 (7.5%)
	Tendinopathy	n = 30 (37.5%)
	Partial tears	n = 31 (38.75%)
	Full-thickness tears	n = 13 (16.2%)

In our study, six patients (7.5%) did not have any injury to the supraspinatus tendon, 30 (37.5%) presented tendinopathy of the supraspinatus, 31 (38.75%) had partial tears and 13 (16.2%) had full-thickness tears (Table 1). We evaluated the prevalence of supraspinatus tears according to the patients’ age group, and we observed that only 21.7% of the patients aged ≤ 40 years had these lesions, while 78.3% of the patients in this age group did not present tears of this tendon; among the patients over 40 years of age, 68.4% had supraspinatus tears. We also found that the female participants presented higher prevalence (65.5%) than the males (49%); and that the left side (57.6%) and the non-dominant side (63.0%) were mostly affected (Table 2).

**Table 2:** Prevalence of supraspinatus tears as detected using magnetic resonance imaging (MRI)

Supraspinatus tears	Yes		No	
	n	%	n	%
≤ 40 years	5	21.7	18	78.3
> 40 years	39	68.4	18	31.6
Male	25	49.0	26	51.0
Female	19	65.5	10	34.5
Right side	25	53.2	22	46.8
Left side	19	57.6	14	42.4
Dominant side	27	50.9	26	49.1
Non-dominant side	17	63.0	10	37.0

Table 3 shows the calculations of sensitivity, specificity, positive predictive value, negative predictive value, accuracy, positive likelihood ratio and negative likelihood ratio.

**Table 3:** Accuracy of ultrasonographic examination

		Magnetic resonance imaging				
			Yes	No	Total	P- value
Ultrasonography	All tears	Yes	16	3	19	0.003
		No	28	33	61	
		Total	44	36	80	
		Sensitivity = 36.3%; specificity = 91.7%; PPV = 54.1%; NPV = 84.2%; accuracy = 61.3%; PLR = 4.36; NLR = 0.69				
	Partial tears	Yes	8	4	12	0.031
		No	23	45	68	
		Total	31	49	80	
		Sensitivity = 25,8%; specificity = 91.8%; PPV = 66.7%; NPV = 66.2%; accuracy = 66.3%; PLR = 3.16; NLR = 0.8				
	Full-thickness tears	Yes	6	0	6	< 0.001
		No	7	67	74	
		Total	13	67	80	
		Sensitivity = 46.2%; specificity = 100%; PPV = 100%; NPV = 90.5%; accuracy = 91.3%; PLR = n.c.; NLR = 0.53				
	Tendinopathy	Yes	16	21	37	0.325
		No	14	29	43	
		Total	30	50	80	
		Sensitivity = 53.3%; specificity = 58%; PPV = 43.2%; NPV = 67.4%; accuracy = 56.2%; PLR = 1.26; NLR = 0.8				
	All lesions (tears and tendinopathy)	Yes	16	3	19	0.002
		No	28	33	61	
		Total	44	36	80	
		Sensitivity = 36.4%; specificity = 91.7%; PPV = 84.2%; NPV = 54.1%; accuracy = 61.3%; PLR = 4.36; NLR = 0.69				

PPV = positive predictive value; NPV = negative predictive value; PLR = positive likelihood ratio; n.c. = not calculated; NLR = negative likelihood ratio.

Ultrasonography showed sensitivity of 36.3% and specificity of 91.7% for supraspinatus tears overall: sensitivity of 25.8% and specificity of 91.8% for partial tears and sensitivity of 46.2% and specificity of 100% for full-thickness tears. The sensitivity and specificity for tendinopathy were 53.3% and 58.0%, respectively; and for all lesions (tears and tendinopathy) of the supraspinatus, the sensitivity was 36.4% and the specificity was 91.7%. The highest accuracy and specificity were seen for full-thickness tears (91.3% and 100%, respectively). 

The positive likelihood ratios (PLRs) for tears, partial tears, tendinopathy and all lesions (tears and tendinopathy) were respectively 4.36, 3.16, 1.26 and 4.36. We also calculated the negative likelihood ratios (NLRs) for all tears, partial tears, full-thickness tears, tendinopathy and all lesions: 0.69, 0.80, 0.53, 0.80 and 0.69 (Table 3).

## DISCUSSION

The etiology of rotator cuff tears is multifactorial, and age-related tendon degeneration is the most important risk factor associated with these lesions.^3^^,^^7^ In our study, we observed that the frequency of supraspinatus tears became higher with increasing age among the patients: 48.7% of the patients over 40 years of age presented lesions while only 6.2% of the patients aged 40 years or younger had these lesions.

Male patients presented higher prevalence of lesions (31.2%), and the right and dominant sides were more affected (31.2% and 33.7%, respectively). These results are concordant with those from previous accuracy studies and epidemiological studies and thus demonstrate that the sample was representative.^3^^,^^4^

Evaluation of the accuracy of shoulder ultrasonography for diagnosing these lesions allowed us to compare our results with those found in the worldwide literature. The present study assessed the sensitivity and specificity of ultrasonography for diagnosing supraspinatus tendon injuries, using magnetic resonance imaging as the reference method. We conducted a prospective accuracy study in which 80 patients with shoulder pain were evaluated and, of these, 38.75% presented partial tears and 16.2% presented full-thickness tears. Partial tears were more common in our study, with prevalence almost three times greater than that of full-thickness tears.

We found that ultrasonography had low sensitivity for diagnosing supraspinatus lesions. However, it had high specificity for tears overall (both partial tears and full-thickness tears), such that the ultrasound results were negative in more than 90% of cases of patients without supraspinatus tears. Among patients with tears, we found a high positive likelihood ratio, thus showing that it was four times more likely to find a tear through ultrasonography in patients who really had such lesions than in those who really did not have them.

Regarding full-thickness lesions, we found high accuracy (91.3%), with specificity and positive predictive value of 100%. This shows that ultrasonography is an excellent examination for diagnosing complete rupture of the supraspinatus tendon. Regarding tendinopathy, ultrasound was not a good examination for diagnosing it, with low sensitivity, specificity and positive and negative likelihood ratios.

A systematic review published by Dinnes et al. showed that despite the heterogeneous results in the 38 studies evaluated (total of 2435 patients), ultrasonography showed high sensitivity (S = 0.87; 95% confidence interval, CI: 0.84-0.89) and specificity (SP = 0.96; 95% CI: 0.94-0.97) in making the diagnosis of full-thickness tears of the supraspinatus tendon. For partial tears, ultrasonography showed lower sensitivity (S = 0.67; 95% CI: 0.61-0.73) but higher specificity (SP = 0.94; 95% CI: 0.92-0.96), compared with magnetic resonance imaging. Those authors concluded that ultrasonography and magnetic resonance imaging were equivalent in making the diagnosis of complete rupture of the rotator cuff, and that a negative result from ultrasonography was more likely to rule out a rotator cuff injury than was magnetic resonance imaging.^9^ In our study, we found that ultrasonography had high specificity for both partial and complete supraspinatus tears, but that it had low sensitivity for diagnosing tendon lesions. In the systematic review by Dinnes et al., retrospective studies were also included, and lower sensitivity scores were found for the prospective studies included in the review than for the retrospective studies. Retrospective studies present implicit forms of bias, such as selection and information biases, which relate to the way in which the information was collected. This feature of retrospective studies may lead to results that differ from those of prospective studies. In addition, in the systematic review by Dinnes et al., some studies were carried out in specific radiology centers, using data gathered by radiologists who were specialists in musculoskeletal disorders. This may be one of the reasons for the difference in sensitivity found between our study and the systematic review by Dinnes et al.^9^

Magnetic resonance imaging has sensitivity and specificity that range from 84% to 96%. It is the reference test used in clinical practice for diagnosing ruptures of the rotator cuff. In two systematic reviews on the accuracy of ultrasonography for diagnosing rotator cuff lesions, published by Lenza et al. and Roy et al., it was concluded that ultrasonography and magnetic resonance imaging presented similar accuracy for diagnosing full-thickness tears and low sensitivity for diagnosing partial ruptures of the rotator cuff.^8^^,^^10^

Some studies published on this subject evaluated the accuracy of ultrasonography only among patients with a diagnosis of supraspinatus tears.^7^^,^^11^ In our study, we also evaluated patients with no lesions and with tendinopathy of this tendon and we calculated the accuracy of ultrasonography for making the diagnosis of supraspinatus tendinopathy (accuracy = 56.2%), i.e. not only for partial and total tears.

In our study, the analysis was performed based on a sample of patients who had shoulder pain. In addition, the reports on the examinations were made by several evaluators (five radiologists) who were not specialists in musculoskeletal disorders, thereby increasing the external validity of our study. The limitations of our study were that the data were collected in a single center and the number of patients was small (N = 80), among whom only 13 presented complete rupture of the supraspinatus. We did not use arthroscopy as a reference test (magnetic resonance imaging was used); and the level of interobserver agreement between the evaluators was not calculated. However, despite these limitations, we found results regarding the accuracy and specificity of complete supraspinatus rupture that were similar to what has been reported in the published literature.

It is common in our practice to find situations in which, for example, an ultrasound examination may show tendinopathy while magnetic resonance imaging on the same patient may show a rotator cuff tear. Such situations can be explained by the low sensitivity found in our study. They may occur because it is infrequent in our setting for radiology centers to have radiologists who are also specialists in musculoskeletal disorders, for performing ultrasound examinations. Such specialists would have greater training and would be accustomed to performing ultrasound examinations of the shoulder. Our situation thus differed from the situation of the studies included in the systematic review conducted by Dinnes et al.^9^

Accuracy studies provide the best evidence for making a diagnostic evaluation on a test or examination. However, the lack of standardization found in older studies is one of the reasons for the variability of the published results. We conducted a standardized prospective accuracy study on ultrasound examinations, and we found high accuracy in making the diagnosis of full-thickness tears of the supraspinatus tendon. However, ultrasonography presented low sensitivity for detecting lesions of this tendon. We therefore recommend that further studies including larger numbers of patients should be conducted to evaluate the sensitivity and specificity of ultrasonography for diagnosing injuries of the supraspinatus tendon and other tendons.^12^

## CONCLUSION

Ultrasonography showed low sensitivity for detecting supraspinatus tears, but high specificity for both partial and full-thickness tears.
